# Structural Position Correlation Analysis (SPCA) for Protein Family

**DOI:** 10.1371/journal.pone.0028206

**Published:** 2011-12-05

**Authors:** Qi-Shi Du, Jian-Zong Meng, Cheng-Hua Wang, Si-Yu Long, Ri-Bo Huang

**Affiliations:** 1 State Key Laboratory of Non-food Biomass Energy and Enzyme Technology, National Engineering Research Center for Non-food Biorefinery, Guangxi Academy of Sciences, Nanning, Guangxi, China; 2 Life Science and Biotechnology College, State Key Laboratory for Conservation and Utilization of Subtropical Agro-bioresources, Guangxi University, Nanning, Guangxi, China; 3 Biotechnology and Pharmaceutical Engineering, Nanjing University of Technology, Nanjing, Jiangsu, China; Russian Academy of Sciences, Institute for Biological Instrumentation, Russian Federation

## Abstract

**Background:**

The proteins in a family, which perform the similar biological functions, may have very different amino acid composition, but they must share the similar 3D structures, and keep a stable central region. In the conservative structure region similar biological functions are performed by two or three catalytic residues with the collaboration of several functional residues at key positions. Communication signals are conducted in a position network, adjusting the biological functions in the protein family.

**Methodology:**

A computational approach, namely structural position correlation analysis (SPCA), is developed to analyze the correlation relationship between structural segments (or positions). The basic hypothesis of SPCA is that in a protein family the structural conservation is more important than the sequence conservation, and the local structural changes may contain information of biology functional evolution. A standard protein P^(0)^ is defined in a protein family, which consists of the most-frequent amino acids and takes the average structure of the protein family. The foundational variables of SPCA is the structural position displacements between the standard protein P^(0)^ and individual proteins P*_i_* of the family. The structural positions are organized as segments, which are the stable units in structural displacements of the protein family. The biological function differences of protein members are determined by the position structural displacements of individual protein P*_i_* to the standard protein P^(0)^. Correlation analysis is used to analyze the communication network among segments.

**Conclusions:**

The structural position correlation analysis (SPCA) is able to find the correlation relationship among the structural segments (or positions) in a protein family, which cannot be detected by the amino acid sequence and frequency-based methods. The functional communication network among the structural segments (or positions) in protein family, revealed by SPCA approach, well illustrate the distantly allosteric interactions, and contains valuable information for protein engineering study.

## Introduction

It is commonly accepted that the evolution of a protein family is the result of large-scale random mutagenesis of amino acids, with selection constraints imposed by their biological functions. Correspondingly most existing computational methods for prediction of functional evolution of protein families are designed based on the statistical analysis of amino acid sequences of the protein family. This type approaches begin from a database of multiple sequence alignment in the protein family, then amino acid frequencies at each sequence position are calculated, which is the fundamental quantity in the statistical analysis of protein evolutionary family [1]–[4].

Long time ago scientists had noticed that the individual proteins in a protein family, which perform the similar biological function, may have very different amino acid composition, but they must share the similar three dimensional structure, and keep a stable key structural region [5]. In other words, sharing the similar structural folding pattern is the necessary condition for all members in a protein family. Therefore the structural conservation is more important than the conservation of amino acid composition. The α-amylase protein family is a good example, which has an average sequence length of 420 amino acids. Among the 420 amino acids only 8 to 10 residues are absolutely conservative, and all other residues may be different more or less [6]. On the other hand, the proteins of α-amylase family have a very conservative structure region, TIM (β/α)_8_ barrel, and all other structural regions may be different.

The differences in biological activity of individual proteins in a family are determined not only by the mutations of amino acids, but also by the structural differences. For example, all types of neuraminidases (NA) of influenza A viruses, which is the drug target of oseltamivir [7] and zanamivir [8], share the same folding pattern of 3D structures. However, small structural difference at 150-loop in NA subtypes may cause the drug resistant problem [9]. On the other hand, the structural differences at 150-loop of NA subtypes are the structural basis for designing effective drugs against specific subtype of influenza virus [10].

In the previous studies of statistical analysis for functional evolution of protein family, most attentions had focused on the amino acid conservation and mutation [11]–[14]. In this study a computational approach, namely structural position correlation analysis (SPCA), is developed to predict mutual correlations of structural segments and positions, and to find the signal communication network in protein family. We expect that the SPCA approach may find applications in protein engineering and in structure-based rational drug design.

## Results

To test the effectiveness of the SPCA theory and method, developed in this study, the PDZ domain family is selected as a model system, which is a well studied protein family [15]–[18].

### Database of PDZ protein domain

**Figure 1:**
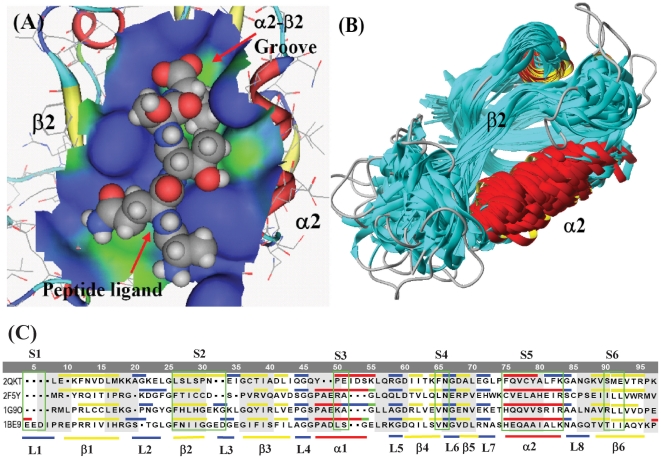
Structure of PDZ domain 1BE9 and multiple structural alignment (MSA) of 186 PDZ domains. (**A**) The structure of PDZ domain 1BE9 and peptide ligand. Target C-terminal ligands bind in a surface groove formed between the α2 helix and the β2 strand at a number of binding sites that determine both ligand affinity and sequence specific recognition. Blue is for hydrophilic surface and green for hydrophobic surface. (**B**) The multiple structural alignment (MSA) database of 186 PDZ crystal structures. PDZ domains consist of 90–100 residues that adopt a six-stranded β sandwich configuration with two flanking α helices. In the MSA database there are 117 residue positions, including gaps inserted in structural alignment. After deletion of unnecessary gaps, the length of MSA database is 96 positions. (**C**) The locations of 6 structural segments and the secondary structural units of PDZ protein domains. The four PDZ protein domains (2QKT, 2F5Y, 1G9O, and 1BE9) are taken from the MSA database of 186 PDZ domains. The six structural segments (S1 to S6) are indicated by green frameworks, and the secondary structural units (α-helices, β-strands, and loops) are indicated by color bars (blue for loops, yellow for β-strands, and red for α-helices). The structural segments are stable units in the structural changes of protein family.

The PDZ is a common structural domain found in the signaling proteins of bacteria, yeast, plants, viruses [19], [20], animals [21], [22], and human [23]. The PDZ domains consist of 90–100 amino acid residues that adopt a six-stranded β sandwich configuration with two flanking α helices. The structure of PDZ domain 1BE9 and peptide ligand is shown in Fig. 1 A.

In the PDZ domain the target C-terminal ligands bind in a surface groove formed between the α2 helix and the β2 strand at a number of binding sites that determine both ligand affinity and sequence specific recognition [24], [25]. Both the overall three-dimensional structure and most details of ligand recognition are highly conserved in the family despite considerable sequence divergence [26]. The PDZ domains well represent protein binding motifs for which four high-resolution structures of distantly related members exist [24], [27], [28]. These domains help anchor transmembrane proteins to the cytoskeleton and hold together signaling complexes [29].

In this study the multiple structural alignment database consists of 186 3D structures of PDZ protein domains, which are selected from protein data bank (http://www.rcsb.org/pdb/). After structural sequence alignment there are 117 residue positions, and after deletion of the unnecessary gaps, the length of database is reduced to 96 positions. The MSA structural alignment of 186 PDZ domains is shown in Fig. 1 B.

### Position structural displacement matrix

**Figure 2:**
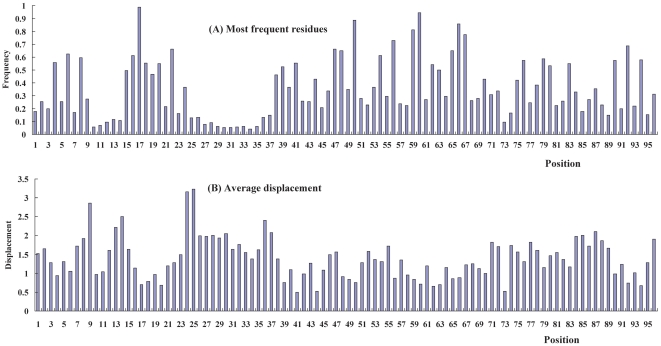
The most frequent amino acids at sequence positions and the average position displacements between the standard protein and the proteins of PDZ domains. (**A**) The percent frequencies of the most frequent amino acids at sequence positions of the MSA PDZ domains database. The higher frequency means the higher conservation and the lower frequency means the higher mutation of amino acids at the sequence positions. (**B**) The average structural displacement between standard protein P^(0)^ and the proteins of PDZ domain database. The higher displacement represents the larger structural change at the positions, and the lower displacement indicates the stable positions in structure. Partially complementary relationship between the amino acid frequencies and the structural displacement is found: the higher amino acid frequency, the lower position displacement.

Following the procedure described in method section, the standard protein P^(0)^ and position displacement matrix D^(α)^_L×L_ of the PDZ domain database is built. Fig. 2 A shows the most frequent amino acids at sequence positions, and Fig. 2 B shows the average position displacements between the standard protein P^(0)^ and the proteins of PDZ domains.

In Fig. 2 A the higher frequency represents the stronger conservation of amino acid at the structural positions, and the lower frequency indicates the higher mutation of amino acid at the positions. In Fig. 2 B the higher displacement represents the larger structural change at the positions, and the lower displacement indicates the stable positions in the structural change. In Fig. 2 A there are several positions, at which the amino acids have very high frequencies: G at position 18, A at position 50, G at position 59, D at position 60, N at position 66, and G at position 67. These positions are the most conservative positions and listed in Table 1. Based on the most conservative positions the position displacement matrix D^(α)^_N×L_ and D^(m)^_N×L_ are built in the second MSA step using Eq.5.

**Table 1:** The most conservative positions* in PDZ domain database.

Position	Amino Acid	Frequency	Displacement Å
17	Gly (G)	0.9892	0.7003
50	Ala (A)	0.8871	0.7528
59	Gly (G)	0.8118	0.8452
60	Asp (D)	0.9462	0.7129
66	Asn (N)	0.8602	0.8757
67	Gly (G)	0.8042	0.8862

*The frequency of residue *k* at the most conservative position *l* is larger than 0.80, f^(m)^ *_k,l_*>0.80.

After careful observation at Fig. 2 A and B, we find interesting complementary relationship between the amino acid frequencies and the structural displacements: the higher amino acid frequency, the lower position structural displacement. All the most conservative positions have very small position displacements, as shown in Table 1. Correspondingly in Fig. 2 B at these positions the structural displacements are small. In Fig. 2 A at the positions from 25 to 37 the amino acid frequencies are very small. In contrast in Fig. 2 B the structural displacement at these positions are high. As we know that the amino acid position frequency is the fundamental quantity in the statistical coupling analysis (SCA) [11]–[14] and CMCA (conservation-mutation correlation analysis) [30]. According to the complementary relationship between amino acid position frequencies and position structural displacements, we expect that the structural position correlation analysis (SPCA) may provide useful information from different aspects to the functional evolution study of protein family.

### Structural segments of PDZ domains

From the position structural displacement matrix D^(α)^_L×L_ of the PDZ domain database and using the Eq.7 to Eq.9, we get the position displacement correlation matrix R^(α)^_L×L_. From the calculation results we find high correlation coefficients among some continuing sequence positions. The correlation coefficients, higher than 0.60, are listed in Table 2. The positions in Table 2 fall in 6 segments: positions 4 to 7 in segment S1, positions 26 to 34 in segment S2, positions 50 and 51 in segment S3, positions 65 and 66 in segment S4, positions 75 to 83 in segment S5, and positions 90 to 92 in segment S6. For convenience in this study only the segments consisting of 2 or more positions are called segments and numbered as S*_k_*.

**Table 2:** The large position displacement correlation coefficients (*r_i,j_*>0.60) in the PDZ domain database.

Position		Coefficient	Position		Coefficient
*i*	*j*	*r_i_*_,*j*_	*i*	*j*	*r_i_*_,*j*_
4	5	0.6110	34	32	0.7807
4	6	0.7217	34	33	0.8587
5	4	0.6110	50	51	0.8339
5	6	0.6163	51	50	0.8339
6	4	0.7217	65	66	0.6319
6	5	0.6163	66	65	0.6319
6	7	0.6017	75	76	0.6251
7	6	0.6017	75	77	0.6207
26	27	0.7769	76	75	0.6251
26	28	0.7058	76	77	0.7074
27	26	0.7769	76	79	0.6524
27	28	0.8039	76	80	0.6704
27	29	0.6893	77	75	0.6207
28	26	0.7058	77	76	0.7074
28	27	0.8039	77	78	0.6508
28	29	0.7083	77	79	0.6543
28	30	0.6280	77	80	0.6850
29	27	0.6893	78	77	0.6508
29	28	0.7083	78	79	0.7340
29	30	0.8635	79	76	0.6524
29	31	0.6595	79	77	0.6543
30	28	0.6280	79	78	0.7340
30	29	0.8635	79	80	0.6941
30	31	0.8007	79	82	0.6015
30	32	0.6263	79	83	0.7306
31	29	0.6595	80	76	0.6704
31	30	0.8007	80	77	0.6850
31	32	0.7713	80	79	0.6941
31	33	0.6857	80	81	0.6937
32	30	0.6263	80	83	0.7206
32	31	0.7713	81	80	0.6937
32	33	0.9105	82	79	0.6015
32	34	0.7807	83	79	0.7306
33	31	0.6857	83	80	0.7206
33	32	0.9105	90	92	0.6048
33	34	0.8587	92	90	0.6048

The PDZ domain consists of six β-strands, two α-helices, and eight loops. There are certain relationship between structural segments and secondary structural units. The segment 1 (S1) is located in the loop 1 (L1), the S2 is in the β2 and foreside of loop L2, S3 is in α1, S4 covers part of β4 and part of L6, S5 is basically in α2, and S6 is in β6. The sequence alignment of four PDZ domains (2QKT, 2F5Y, 1G9O, and 1BE9) is shown in Fig. 2 C. The relationship between 6 structural segments and secondary structural units of PDZ domain database is indicated in Fig. 2 C.

In the 6 structural segments there are 29 positions. Except the 29 positions in 6 segments, the other positions are independent segments (positions). Therefore, in the PDZ domain database the number of segments is K = 73. The segment displacement matrix D^(s)^_K×K_ is calculated using Eq.5. Then the segment displacement covariance matrix C^(s)^_K×K_ and correlation matrix R^(s)^_K×K_ are calculated using Eq.7 to Eq.9, respectively.

**Figure 3:**
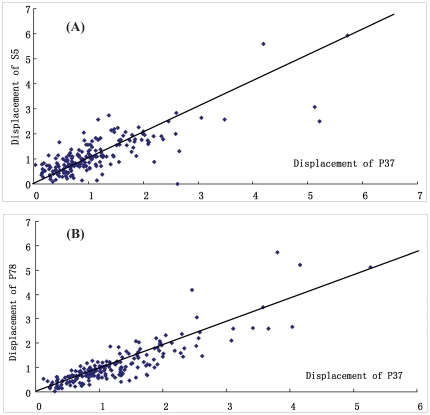
The displacement correlation relationships between structural segments and positions. (**A**) The displacement correlation between segments S2 (in β2) and S5 (in α2). The correlation of S2 and S5, actually, represents the structural correlation between α2 and β2. (**B**) The displacement correlation between position 37 (in β3) and position 78 (α2). The correlation of positions 37 and 78 causes a distant allosteric interaction in the PDZ domain.

From the segment displacement correlation coefficients R^(s)^_K×K_ we find the correlation relationship among the structural segments and positions of PDZ domains. As shown in Fig. 3 A, the displacement of structural segment S2 is intensely correlated with that of the segment S5, and the higher correlation relation between position 37 in β3 and position 78 in α2 is shown in Fig. 3 B.

### Information abstraction of PDZ domain

**Figure 4:**
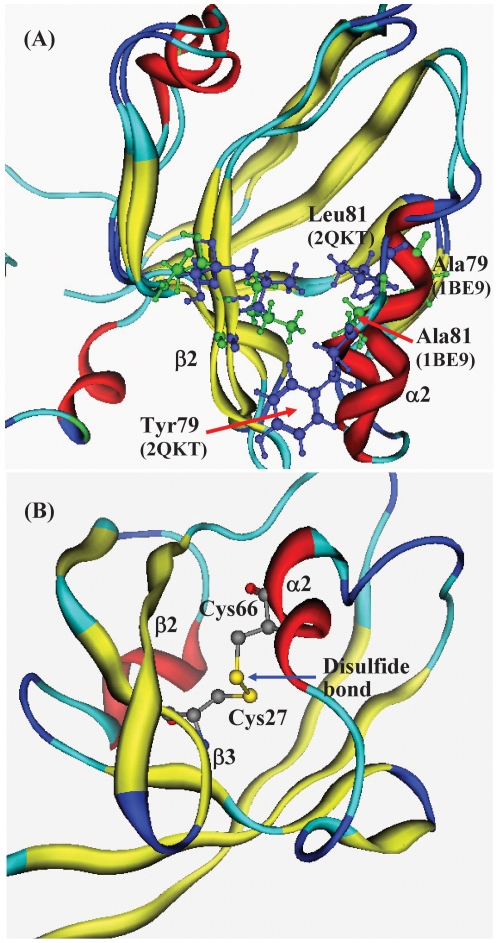
Information for PDZ protein domain from the SPCA calculation. (**A**) The residues at the controlling positions for ligand affinity. The size of Tyr79 and Leu81 of 2QKT (blue) are much larger than the Ala76 and Ala78 of 1BE9 (green). (**B**) The disulfide bond between Cys37 in β3 and Cys78 in α2 of 2QKT. The interaction between positions 37 and 78 indirectly conducts the controlling signal to the ligand preference of binding location in α2-β2 groove.

Some useful information for functional evolution study of PDZ domain family is abstracted from the calculation results of SPCA calculation. The groove between α2 helix and β2 strand is the binding location for peptide ligand [12]. Amino acid mutations and structural changes at these positions play important roles to the functional difference of PDZ domains. As shown in Fig. 3 A, the structural displacement of S2 (in β2) is intensively correlated with S5 (in α2). The structural correlation between α2 and β2 well illustrates the ligand affinity and recognition specificity of PDZ domains to the peptide ligands. Fig. 4 A shows the α2-β2 groove of PDZ domains 1BE9 and 2QKT. Experiments found that in α2-β2 groove there are some easily mutative positions: 79 and 81 in α2, and 27 and 28 in β2 (in Fig. 1 C numbering), which determine the ligand binding affinity and control the shape and physicochemical property of the peptide ligands. In Fig. 4 A the residues Ala79 and Ala81 (in green) of 1BE9 are replaced by residues Tyr79 and Leu81 (in blue) of 2QKT. The size of Tyr79 and Leu81 of 2QKT are much larger than the Ala79 and Ala81 of 1BE9. Therefore 1BE9 and 2QKT must have very different preferences for peptide ligands. The correlation of amino acid mutations at these positions between α2 and β2 is accompanied by the correlation between structural displacement of segments S2 and S5, hence affects the preference of peptide ligands.

The mechanism of distance allosteric interaction in proteins is a challenge and open research topic [31]. The protein functions are not only determined by the interactions between local residues, but also depend on nonlocal and long-range communication between amino acids [32]. For example, allosteric regulation in various proteins [33], [34], the distributed dynamics of amino acids involved in enzyme catalysis [35]–[37], and information transmission between distant functional surfaces on signaling proteins [38], all represent manifestations of nonlocal interactions between residues.

Long-range allosteric effects that cause the preference change of peptide ligands in the PDZ binding groove were found in several distant positions [39]. In the alignment of four PDZ proteins in Fig. 1 C the 2QKT [40] is an INAD PDZ domain [41] and belongs to type 5 PDZ. The INAD PDZ domain (PDZ5) exists in a redox-dependent equilibrium [42], [43] between an oxidized form and a reduced form. In the INAD PDZ an intramolecular disulfide bond covalently links a pair of buried cysteine residues located below the floor of the ligand-binding pocket [39], [44], as shown in Fig. 4 B. In 2QKT the disulfide bond is formed between Cys37 in β3 and Cys78 in α2 (in Fig. 1 C numbering). The positions of Cys37 and Cys78 are corresponding to the residues Ile37 and Ala78 (in Fig. 1 C numbering) of 1BE9, respectively.

The correlation of structural displacement between position 37 and 78 gives a good explanation to the distance allosteric interaction of mutations at β3 to the ligand preference of α2-β2 groove. The interaction between positions 37 and 78 affects the connection between β3 and α2, therefore, causes the structural change of the α2-β2 groove indirectly, hereby change the ligand preference of PDZ domains indirectly.

## Discussion

Structural conservation is the necessary condition for all members of a protein family, and the local structure differences may be responsible for the functional differences of individual proteins. Taking the structural data into the consideration of statistical analysis for protein evolutionary family certainly can find useful information that cannot be revealed by the amino acid sequence and frequency-based methods.

The theoretical implications of SPCA approach are summarized as follows. (i) The standard protein P^(0)^ of a protein family, in which the position coordinates are the average coordinates of corresponding residues of all proteins and the residues at each position are the most frequent amino acid, keeps the common structural features of the family that are shared by all protein members. (ii) The most conservative positions form the structural core, and the amino acids at the most conservative positions perform the biological activity. The residues at other positions provide the physicochemical environment for the functional residues. The influences of non functional residues to the functional residues are determined not only by the amino acid types, but also by their position displacements. (iii) The position structural displacements between individual protein P*_i_* and the standard protein P^(0)^ are the foundational variables, which determine the bioactivity differences of individual proteins in the family. (iv) The structural segments are the stable structure units of protein family, and the correlation between structural segments (or positions) may conduct signal for distance allosteric interaction.

The application example of PDZ domain proves that the structural position correlation analysis (SPCA) is able to find the correlation relationship among the structural segments (or positions) in a protein family, which cannot be detected by the amino acid sequence and frequency-based methods. The functional communication network among the structural segments (or positions) in protein family, revealed by SPCA approach, well illustrate the distantly allosteric interactions, and contains valuable information for protein engineering and protein design study.

## Methods

Homologous proteins have conservative three dimensional structures that are evolutionarily more conserved than expected due to sequence conservation [45], [46]. The structural position correlation analysis (SPCA) for protein family starts from multiple 3D structural alignment of a protein family.

### Structure alignment and the most conservative positions

The database of SPCA is built in a two-step procedure. The first step is a standard multiple structural alignment (MSA) of the protein family. In the standard MSA the α-carbon coordinates of all residues are realigned taking into account their structural similarity. From the initial estimate of the alignment, a new similarity matrix is generated using the relative α-carbon coordinates that result from a multi-body superposition. This matrix is used to realign just these alpha carbon populated chains. This procedure is then repeated until the root mean square distance (RMSD) of the superposition fails to improve. The multiple structural alignment of an evolutionary protein family reveals the structural features of family: all key functional residues are aligned in the same sequence positions, and all key secondary structures (α-helices, β-sheets, and loops) are positioned in the same sectors.

After the standard multiple structural alignment the composition of protein family is represented by a binary data matrix A_N×M×L_, where N is the number of proteins in the database, M is the types of amino acids (M = 21, including 20 natural amino acids and the gap, which is inserted during the multiple alignment), and L is the length of amino acid sequences (including gaps). In the composition matrix A_N×M×L_ the element *a_i,k,l_* is 1 when the amino acid *k* of protein *i* is at the position *l*, otherwise, it is 0,
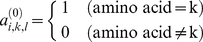
(1)The amino acid position frequency matrix F_M×L_ is constructed from the composition data matrix A_N×M×L_ as follows,
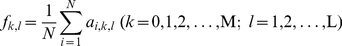
(2)The *f_k,l_* is a decimal value in region [0,1]. The higher value of *f_k,l_* means the higher frequency of amino acid *k* at position *l*. In this study the gaps are treated as a special amino acid type numbered by 0, and the 20 natural amino acids are numbered from 1 to 20. The summation of *f_k,l_* from *k* = 0 to M is 1. At each position *l* the most frequent amino acid *k* is defined as the amino acid that possesses the largest frequency *f* ^(m)^ *_k,l_* at position *l*. The most frequent amino acids {*f* ^(m)^ *_k,l_*, *l* = 1,2,…L} compose the amino acid sequence of standard protein P^(0)^.

In the second step of MSA, a set of most conservative structure positions {*l* ^(m)^ *_j_*} is selected firstly as follows. If the value *f* ^(m)^ *_k,l_* of the most frequent amino acid *k* at position *l* is larger than 0.80 (*f* ^(m)^ *_k,l_*>0.80), the position *l* is the most conservative position. Then the second multiple structural alignment is performed only to the most conservative positions, making the coordinate RMSD of all most conservative positions as smaller as possible. In this way we get the structural database X_N×L_, Y_N×L_ and Z_N×L_ of the protein evolutionary family for the SPCA calculation, in which the elements *x_i,l_*, *y_i,l_* and *z_i,l_* are the Cartesian coordinates of position *l* in protein *i*.

The theoretical consideration of the SPCA database can be illustrated as follows. The residues at most conservative positions are the functional residues, which perform the biological activity. The residues at other positions are non-functional residues, forming the physicochemical environment for the functional residues. The effect of the non-functional residues to the functional residues is determined not only by amino acid types, but also by their structural positions.

### Standard protein of protein family

In a protein family the standard protein P^(0)^ is defined as follows. The amino acid sequence of standard protein consists of the most frequent amino acids at each position, and its 3D structure is the average structure of all proteins in the family. From the SPCA database the structure of standard protein P^(0)^ of the protein family is calculated using the following equations,
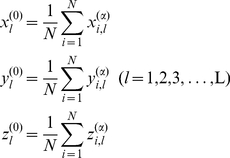
(3)where N is the number of proteins in family, L is the sequence length of MSA database, the superscript α indicates the coordinate of α-carbon of residues, and ‘0’ denotes the standard protein. The standard protein is the common representative of the protein family.

### Displacement matrix of structural positions

The displacement matrix D_N×L_ of protein residue positions is derived from the standard protein and the MSA database of the protein family. The element *d_i,l_* is the distances between the residue *l* of the standard protein P^(0)^ and the residue *l* of protein P*_i_*. There are two types of displacement matrices. One is the distances between α-carbon atoms of standard protein and proteins of family, D^(α)^_N×L_, and the other is the distances of residue mass centers between standard protein and proteins of family, D^(m)^_N×L_. The mass center of residue *l* in protein *i* is computed as follows,
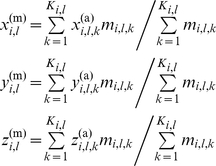
(4)where K*_i,l_* is the number of atoms in residue *l* of protein *i*, x^(α)^ *_i,l,k_* is the cartesian coordinate of atom *k* in residue *l* of protein *i*, and *m_i,l,k_* is the atomic mass of atom *k* in residue *l* of protein *i*.

The elements *d* ^(α)^ *_i,l_* of α-carbon displacement matrix D^(α)^_N×L_ are calculated using the following equation,
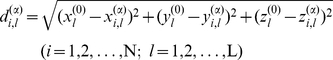
(5)


### Reducing unnecessary gaps

The SPCA calculation is complicated by the presence of alignment gaps inserted in the multiple structural alignment, which is commonly called indels, indicating a structural region present in some proteins but not in others. The gaps (space positions) may interfere with the results of statistical analysis badly. Before performing the correlation analysis we have to reduce the unnecessary gaps. To do so, the total amino acid position frequencies *q_l_* of 20 natural amino acids at each position *l* are needed,
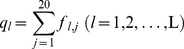
(6)In Eq.6 the index *j* for amino acid types runs from 1 to 20, not including the gap. In the amino acid frequency calculation the gap is a special ‘amino acid’ numbered as 0. If the total amino acid position frequency of the 20 natural amino acids *q_l_* is less than 20%, the position *l* is deleted from the primer MSA database. Because at the position *l* the gaps are more than 80%, the position *l* is less important for the biological function of the protein family. After unnecessary gaps are deleted, the sequence length L is shorter than that of the primer data matrix. For simplicity, we still use L for the reduced sequence length.

### Position displacement correlation

The purpose of SPCA is to find the correlation relationship between structural positions in the protein family. For this purpose we first construct the position covariance matrix C^(α)^_L×L_ from displacement matrix D^(α)^_N×L_ as follows,
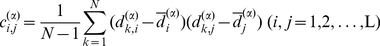
(7)where 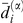 and 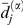 are the average displacements at position *i* and *j*, respectively,
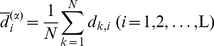
(8)Hereby we get the position displacement correlation matrix R^(α)^_L×L_ from the position covariance matrix C^(α)^_L×L_ as follows,
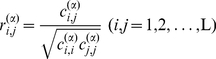
(9)where the superscript ‘α’ indicates the ‘α-carbon’, and *r* ^(α)^ *_i,j_* is the displacement correlation coefficient between position *i* and *j*. In the same way we can calculate the position displacement correlation matrix R^(m)^_L×L_ using mass center displacement matrix D^(m)^_N×L_.

### Fragment displacement correlation

The secondary structures (α-helix, β-strand, and loop) are the structural units of protein structures. In many cases in the structural change of protein family some residues form a relatively stable segment, especially in some secondary structural units. The position structural displacements of the residues in a stable segment are correlated each other strongly. In order to analyze the structural position correlations among the stable segments, especially in the secondary structural units, it is best to organize the residue positions as structural segments. In SPCA a structural segment is defined as a set of continuing positions with higher mutual correlation coefficients (*r* ^(α)^ *_i,j_*>0.60). The coordinates of structural segments are calculated as follows,
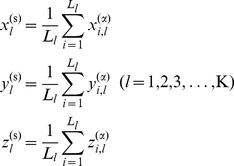
(10)where L*_l_* is the number of positions in segment *l*, the K is the total number of segments, and superscript ‘s’ indicates the segment. The structural segments are not rigorously consistent to the secondary structural units. Some segments may cover continuing residue positions in two secondary structural units. However, many segments may contain only one residue position. The number of structural segments K must be less than the number of residue positions L of the protein family, K<L.

**Figure 5:**
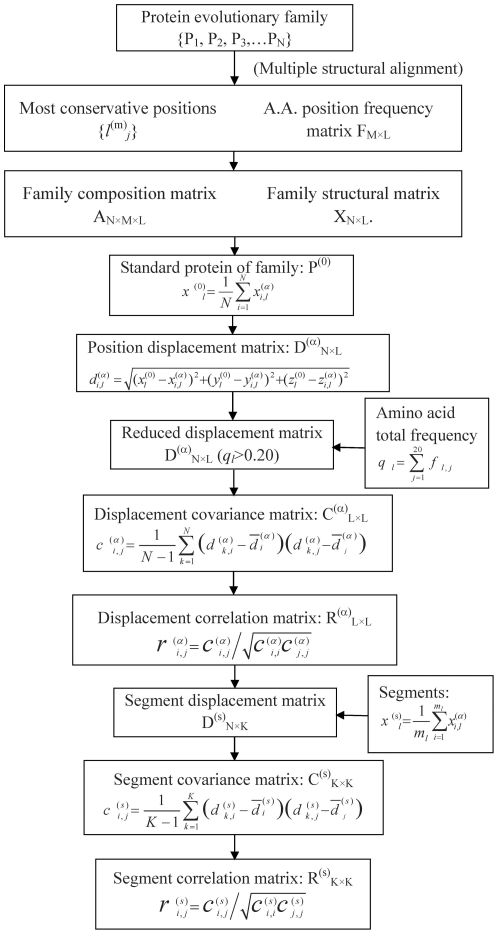
The flowchart of structural position correlation analysis (SPCA). The displacement matrix D^(α)^_N×L_ and D^(m)^_N×L_ is the distant differences between standard protein P^(0)^ and proteins of protein evolutionary family. The superscripts ‘α’ and ‘m’ indicate the α-carbon and mass center, respectively. From the statistical correlation analysis to the residue position displacements D^(α)^_N×L_, the residue positions are reorganized as structural segments. Then statistical correlation analysis is applied to the structural segment displacement matrix D^(s)^_N×K_, revealing the segment correlation information of functional evolution in the protein family.

The displacement matrix D^(s)^_N×K_, the covariance matrix C^(s)^_K×K_, and the segment displacement correlation matrix R^(s)^_K×K_ of structural segments can be calculated using the equations Eq.7, Eq.8, and Eq.9, respectively. The displacement correlation coefficient *r* ^(s)^ *_i,j_* represents the correlation relationship between segments *i* and *j* in protein family. The computational procedure of structural position correlation analysis is graphically illustrated in Fig. 5.
